# The role of platelets in central hubs of inflammation: A literature review: Retraction

**DOI:** 10.1097/MD.0000000000046765

**Published:** 2025-11-21

**Authors:** Yan Bo, Qingyang Lu, Beilei Li, Ren Sha, Haodong Yu, Chuhan Miao

The article, “The role of platelets in central hubs of inflammation: A literature review,”^[1]^ which appears in Volume 103, Issue 19 of *Medicine*, is being retracted. After publication, coauthor Yan Bo contacted the editorial office expressing concern over the publication of an earlier draft of the article that was published as part of the Proceedings of the 2nd International Conference on Biological Engineering and Medical Science^[2]^ and that was not detected by our plagiarism software at the time of acceptance. When contacted, the other authors claimed to have no knowledge of the conference proceeding version.

At the time of submission, each corresponding author is asked to accept and agree to the terms and conditions of our License to Publish agreement. As the corresponding author of the *Medicine* article, Chuhan Miao agreed to our license on behalf of all coauthors, which included the following terms:

(iv) if the Work is a multi-authored Work, the Author has obtained written permission from each author of the Work to enter into this License on behalf such author, and each such author has read, understands and has agreed to the terms of this License;
(vi) neither the Work nor any content contained in the Work, in whole or in part, has been published or is being considered for publication other than in the Journal (as defined in Section 3.a.);
(vii) the Work is not subject to any rights of copyright other than the copyright of the Author and each other author of the Work.

The corresponding author did not disclose the earlier publication or obtain the necessary permission from Yan Bo, who is the copyright holder of the earlier work, which violates the terms of the agreement. All authors have been notified of this retraction.
